# Prospective evaluation of long-term safety of dual-release hydrocortisone replacement administered once daily in patients with adrenal insufficiency

**DOI:** 10.1530/EJE-14-0327

**Published:** 2014-09

**Authors:** A G Nilsson, C Marelli, D Fitts, R Bergthorsdottir, P Burman, P Dahlqvist, B Ekman, B Edén Engström, T Olsson, O Ragnarsson, M Ryberg, J Wahlberg, H Lennernäs, S Skrtic, G Johannsson

**Affiliations:** 1 Department of Endocrinology, Sahlgrenska Academy, University of Gothenburg, Sahlgrenska University Hospital, Gröna Stråket 8SE-413 45, Gothenburg, Sweden; 1 Medical Affairs, ViroPharma SPRL, Maidenhead, UK; 2 Biostatistics ViroPharma Incorporated, Skånes University Hospital, SE-205 02, Malmö, Sweden; 3 Department of Endocrinology, Skånes University Hospital, SE-205 02, Malmö, Sweden; 4 Department of Public Health and Clinical Medicine, Umeå University, SE-90187, Umeå, Sweden; 5 Section of Endocrinology, Department of Medical and Health Sciences, Faculty of Health Sciences, Linköping University, SE-581 83, Linköping, Sweden; 6 Department of Endocrinology, Diabetes, and Metabolism, University Hospital, Uppsala University, SE-751 85, Uppsala, Sweden; 7 Department of Pharmacy, Uppsala University, SE-751 85, Uppsala, Sweden; 8 Department of Clinical Pharmacology, Sahlgrenska Academy, University of Gothenburg, SE-413 45, Gothenburg, Sweden; 9 AstraZeneca R&D, Mölndal, Sweden

## Abstract

**Objective:**

The objective was to assess the long-term safety profile of dual-release hydrocortisone (DR-HC) in patients with adrenal insufficiency (AI).

**Design:**

Randomised, open-label, crossover trial of DR-HC or thrice-daily hydrocortisone for 3 months each (stage 1) followed by two consecutive, prospective, open-label studies of DR-HC for 6 months (stage 2) and 18 months (stage 3) at five university clinics in Sweden.

**Methods:**

Sixty-four adults with primary AI started stage 1, and an additional 16 entered stage 3. Patients received DR-HC 20–40 mg once daily and hydrocortisone 20–40 mg divided into three daily doses (stage 1 only). Main outcome measures were adverse events (AEs) and intercurrent illness (self-reported hydrocortisone use during illness).

**Results:**

In stage 1, patients had a median 1.5 (range, 1–9) intercurrent illness events with DR-HC and 1.0 (1–8) with thrice-daily hydrocortisone. AEs during stage 1 were not related to the cortisol exposure-time profile. The percentage of patients with one or more AEs during stage 1 (73.4% with DR-HC; 65.6% with thrice-daily hydrocortisone) decreased during stage 2, when all patients received DR-HC (51% in the first 3 months; 54% in the second 3 months). In stages 1–3 combined, 19 patients experienced 27 serious AEs, equating to 18.6 serious AEs/100 patient-years of DR-HC exposure.

**Conclusions:**

This long-term prospective trial is the first to document the safety of DR-HC in patients with primary AI and demonstrates that such treatment is well tolerated during 24 consecutive months of therapy.

## Introduction

Adrenal insufficiency (AI) is a life-threatening disease with 2-year mortality in untreated primary AI (Addison's disease) exceeding 80% (1, 2). Conventional treatment involves cortisol replacement therapy with twice- or thrice-daily oral hydrocortisone (3, 4, 5). Although such treatment has remained essentially unchanged for the past 50 years, lower replacement doses have been introduced after new studies showed that the physiological rate of cortisol secretion in healthy individuals is lower than previously thought (6).

Despite conventional hydrocortisone treatment, patients with AI experience signs and symptoms of hyper- or hypocortisolism. Insomnia, increased appetite, weight gain with truncal obesity, hypertension, hyperglycaemia and reduced bone mineral density are indicative of overtreatment with hydrocortisone, while fatigue, muscle pain, nausea and hypoglycaemia are symptoms of cortisol deficiency. Patients receiving conventional therapy for primary AI, or secondary AI as a result of hypopituitarism, have impaired health-related quality of life (7) and an increased risk of cardiovascular events and life-threatening adrenal crisis (8, 9). Studies have shown that mortality in individuals with primary AI receiving conventional therapy remains more than double than that in the healthy population (10, 11), although another study found that the increase in mortality was only in patients who were diagnosed at a young age (12).

The morbidity and mortality associated with conventional treatment for AI are thought to reflect suboptimal replication of the normal serum cortisol profile, resulting in periods of underexposure and overexposure to cortisol, and inadequate rescue therapy during intercurrent illness or periods of physical or mental stress. In healthy individuals, serum cortisol levels are low during the evening and begin to rise between 0200 and 0400 h, reaching a peak within 1 h of waking, and then decline steadily throughout the day (13). In contrast, twice- or thrice-daily doses of hydrocortisone result in non-physiological peaks and troughs of serum cortisol throughout the day. Thrice-daily therapy has been considered the standard of care in patients with AI (14), as it provides more cortisol coverage during the day than twice-daily dosing. However, thrice-daily dosing causes an extra cortisol peak in the late afternoon, with the potential for overexposure to cortisol. Twice-daily therapy is still common in many countries, probably because of suboptimal patient adherence to thrice-daily dosing (15, 16).

Dual-release hydrocortisone (DR-HC; PLENADREN; ViroPharma, Maidenhead, UK) administered once daily consists of an immediate-release coating surrounding an extended-release core. The dual-release tablet provides high levels of cortisol during the morning, followed by a gradual decrease throughout the day. This results in considerably lower cortisol exposure during the afternoon and evening compared with immediate-release thrice-daily hydrocortisone, thereby mimicking normal cortisol secretion more closely than conventional therapy (17).

In a randomised, active-controlled, crossover trial, patients with primary AI had a reduced incidence of cardiovascular risk factors, improved glucose metabolism, and improved health-related quality of life outcomes while receiving DR-HC compared with when they were receiving thrice-daily hydrocortisone therapy (18). During this study, the proportion of patients experiencing adverse events (AEs) was 73.4% in the DR-HC period and 65.6% in the thrice-daily hydrocortisone period. Total daily doses of hydrocortisone were equivalent between the crossover periods, but total cortisol exposure was 19.4% lower while patients received DR-HC compared with when they received thrice-daily hydrocortisone. The present long-term safety study of DR-HC evaluated whether the difference in the incidence of AEs persisted over time and whether it was related to different levels of exposure to cortisol.

## Subjects and methods

### Study design

**Figure 1:**
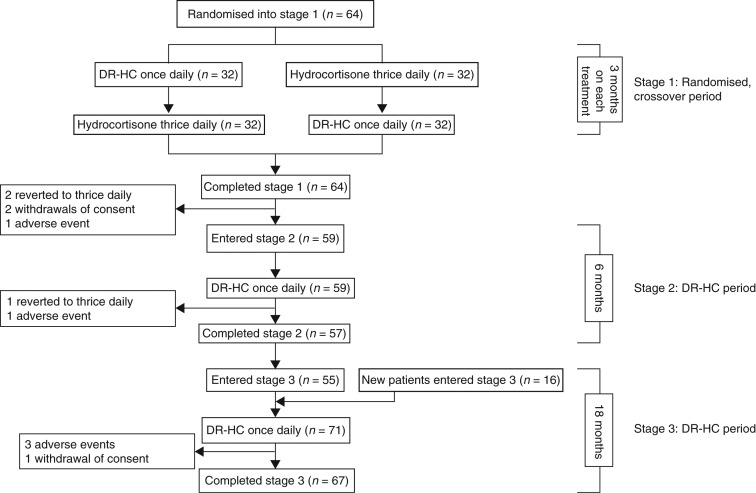
Patient disposition. DR-HC, dual-release hydrocortisone.

The study was conducted in five university clinics in Sweden and occurred in three stages (Fig. 1); the methods of the first two stages have previously been described in detail (18). Briefly, stage 1 was a randomised, open-label, active-control, crossover trial in which patients were randomly assigned to receive either DR-HC or thrice-daily hydrocortisone therapy for 3 months and then crossed over to the other treatment for another 3 months using the same daily dose of hydrocortisone (EudraCT number: 2006-007084-89). All patients received thrice-daily hydrocortisone during a 1-month run-in period before randomisation. Patients who were receiving twice-daily hydrocortisone before the study were switched to thrice-daily hydrocortisone at the beginning of the run-in period.

In stage 2, all patients who participated in stage 1 were eligible to receive open-label DR-HC for an additional period of 6 months (18). Doses of study medication were kept constant throughout stages 1 and 2.

Stage 3 was an open-label extension, in which patients who completed stage 2, and 16 new patients received DR-HC, with dose adjustments allowed at the treating clinician's discretion (EudraCT number: 2008-003990-42). The data from stage 3 reported here are from an 18-month interim analysis. This allowed a prospective analysis of safety of glucocorticoid replacement for a total period of 30 months in patients who entered stage 1. Patients who were initially assigned to receive DR-HC crossed over to thrice-daily hydrocortisone before continuing through stages 2 and 3 on DR-HC for 24 consecutive months (27 months of DR-HC in total). Patients who were initially assigned to receive thrice-daily hydrocortisone, crossed over to DR-HC before continuing through stages 2 and 3 on DR-HC for a total of 27 consecutive months.

Patients returned to the clinic every 4 weeks during phases 1 and 2, and every 3–6 months during phase 3 for studying drug dispensing, AE assessment, and collection of patient questionnaires. Cortisol pharmacokinetic sampling over 24 h was carried out at randomisation and at the end of each 12-week period (multiple-dose pharmacokinetics). A reduced sampling scheme for single-dose pharmacokinetics was performed on day 1 or 2, and multiple-dose pharmacokinetic sampling was performed on day 7 or 8 in 46 patients in both of the 12-week periods as previously described (18). The patients remained at the clinical trial unit on sampling days and received standardised meals.

### Study participants

Patients were eligible for inclusion in the study if they were at least 18 years of age with a diagnosis of primary AI at least 6 months before study entry and had been treated with a total daily hydrocortisone dose (stable for at least 3 months) of 20, 25, 30, 35, or 40 mg. Exclusion criteria included: clinical or laboratory signs of significant cerebral cardiovascular, respiratory, hepato-biliary, or pancreatic diseases that could interfere with study assessments or completion; clinically significant renal dysfunction with a serum creatinine level above 150 mmol/l; any underlying disease possibly requiring treatment with glucocorticoids; and administration of other investigational drugs within 8 weeks before screening. Entry criteria for stage 3 were similar to those of stages 1 and 2, which have been previously described (18). However, any medication or agents interfering with cortisol metabolism was contraindicated in stages 1 and 2, but not in stage 3 (Table 1).

All patients received oral and written study information and provided signed informed consent before entering the study. The study protocols were approved by the Ethics Committee at the Sahlgrenska Academy, Gothenburg and by the Swedish Medical Product Agency. The study was carried out according to the principles of Good Clinical Practice (CPMP/ICH/135/95) and the Declaration of Helsinki.

### Intervention

The patients were instructed to take DR-HC orally once daily at 0800 h as 20 and 5 mg tablets in various combinations to make total daily doses of 20, 25, 30, 35, or 40 mg. In stage 1, the reference drug was hydrocortisone administered orally as 10 mg tablets. The total daily doses were divided into three individual doses administered at 0800, 1200, and 1600 h, with 50–60% of the total daily dose being given as the morning dose. During periods of intercurrent illness, the total daily dose of hydrocortisone or DR-HC was doubled. The patients receiving conventional hydrocortisone therapy doubled the dose at each appointed administration time, and patients receiving DR-HC received a second DR-HC dose 8±2 h after the first dose. The reason for adopting this regimen for DR-HC is that the relationship between cortisol exposure and dose is not proportional (19). Thus, if a single dose is increased from 20 to 40 mg, the serum cortisol exposure is not doubled, whereas the same dose of hydrocortisone administered on two separate occasions will double the exposure.

### Outcomes

Safety was assessed by reporting AEs and periods of intercurrent illness. The patients kept a diary to document extra doses of hydrocortisone or DR-HC and reasons they were taken. A serious AE was defined in accordance with the definition of the World Medical Association, Declaration of Helsinki as any untoward medical occurrence that at any dose results in, for example, death, life-threatening event or in-patient hospitalisation. Patients underwent regular clinical and biochemical examinations throughout the study. The study visits occurred monthly during stage 1, every 3 months during stage 2 and every 6 months during stage 3 (except for patients who were new to stage 3 or who had a visit at baseline, 3 months and every 6 months thereafter).

Serum cortisol was measured in stage 1 using direct chemiluminescence technology (ADVIA Centaur; Bayer Diagnostics), with a sensitivity of 5.5 nmol/l and total coefficient of variation of <8%. Total exposure to cortisol was measured as the area under the curve (AUC) during 24 h (AUC_0–24 h_) and partial time exposure during the afternoon as AUC_6–12 h_.

### Statistical analysis

Statistical analyses were performed using Fisher's exact test to determine differences between treatments in stage 1 and whether the initial increase in AEs during DR-HC was driven by changes in exposure to cortisol. The percentage of AEs in each quintile of serum cortisol exposure (assessed as AUC_0–24 h_ and AUC_6–12 h_) was compared between thrice-daily hydrocortisone and DR-HC. Descriptive data are presented for stages 2 and 3 because they did not include comparator groups.

## Results

### Baseline demographics and disease characteristics

In total, 64 patients were randomised and entered stage 1 (Fig. 1). The mean age of these patients was 47.3 years, approximately half (58.7%) were men and mean BMI was 26.2 kg/m^2^ (Table 1). Eleven patients had diabetes mellitus and 23 were receiving thyroxine replacement. Most patients (*n*=37; 58.7%) were receiving hydrocortisone at a dose of 30 mg/day. Of the 64 patients who entered and completed stage 1, 59 entered stage 2 and 55 entered stage 3 (Fig. 1). In addition, 16 new patients were recruited to stage 3. The mean age and BMI of these new patients were similar to those of patients who entered stage 1, although the proportion of men was lower (31.3%). The patients in this group were generally receiving lower doses of hydrocortisone at study entry than those who entered stage 1 (Table 1).

### Intercurrent illness

During stage 1, when patients received DR-HC or thrice-daily hydrocortisone for 3 months and then crossed over to the other treatment for 3 months, the median number of intercurrent illness episodes per patient was 1.0 while receiving thrice-daily hydrocortisone and 1.5 while receiving DR-HC (Table 2). The median number of days per episode was 2.0 for both treatment periods, and the median extra hydrocortisone dose per episode was 10.0 and 20.0 mg respectively. During 3-month intervals in stages 2 and 3, the median number of intercurrent illness episodes ranged from 1.0 to 2.0, the median number of days per episode ranged from 2.0 to 3.1, and the median extra hydrocortisone dose per episode ranged from 11.7 to 20.0 mg. These values were similar to those of the 16 patients who started in stage 3.

### Serious AEs

When data from stages 1–3 were combined, the results represent up to 27 months of follow-up for the 55 patients who started in stage 1 and continued to stage 3, and up to 18 months of follow-up for the 16 patients who started in stage 3, giving a total of 145 patient-years of exposure to DR-HC treatment. In the combined data set, 19 patients experienced 27 serious AEs while receiving DR-HC, equating to a total of 18.6 serious AEs/100 patient-years of exposure. The rate of serious AEs while patients received thrice-daily hydrocortisone treatment for 3 months in stage 1 was 13.3 events/100 patient-years. Serious AEs while receiving DR-HC were gastroenteritis in 11 patients, adrenal crisis in two patients, and accidental death, acute pancreatitis, cholelithiasis, colitis, ectopic pregnancy, gastritis, hysterectomy, influenza, nephrolithiasis, pneumonia, pyelonephritis, subarachnoid haemorrhage, varicella, and viral infection in one patient each. Sixteen of these serious AEs were infections corresponding to a total of 11.0 events/100 patient-years of exposure. All except one (accidental death) of the serious AE resulted in patient hospitalisation and treatment with parenteral hydrocortisone.

### Long-term safety profile of DR-HC

**Figure 2:**
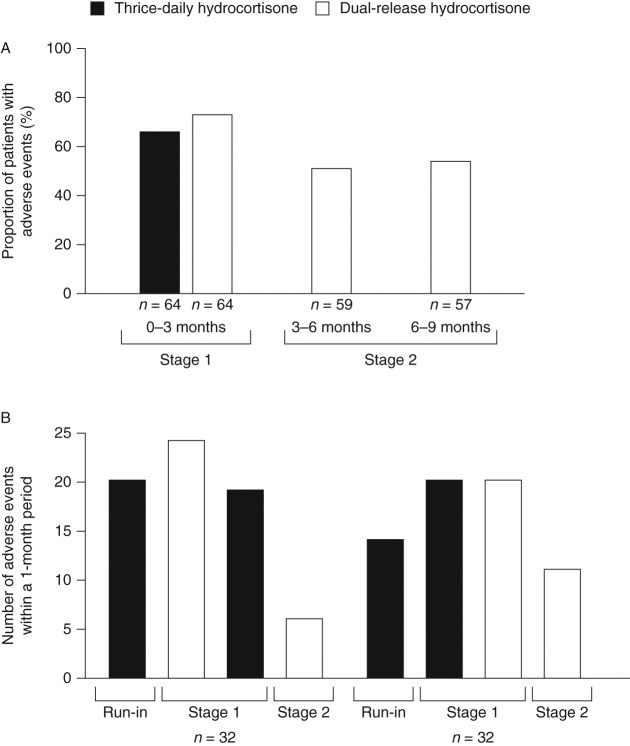
(A) Percentage of patients with at least one adverse event (AE) during 3-month intervals of stages 1 and 2. (B) Total number of AEs that occurred during the 1-month run-in period and the first month after randomisation in stage 1, crossover in stage 1 and entry in stage 2 for patients who were initially assigned to dual-release hydrocortisone (DR-HC; *n*=32) and those who were initially assigned to thrice-daily hydrocortisone (*n*=32).

AE data for stages 1 and 2 have been reported previously (18). During stage 1, the percentage of patients with at least one AE was 65.6% while receiving thrice-daily hydrocortisone and 73.4% while receiving DR-HC (18) (Fig. 2A). During subsequent 3 month intervals in stage 2, the percentage of patients with at least one AE while receiving DR-HC ranged from 51 to 54% (Fig. 2A).

To evaluate the effect of switching treatment on AEs, the total numbers of AEs during the 1-month run-in period, the first 4 weeks after randomisation in stage 1, the first 4 weeks after crossover to the other treatment in stage 1, and the first 4 weeks after entry into stage 2 are presented in Fig. 2B separately for patients who were randomly assigned to DR-HC (*n*=32) or thrice-daily hydrocortisone (*n*=32) during the first period of the crossover. The numbers of AEs during the first 4 weeks after randomisation increased relative to the numbers during the run-in period in both patients who were randomly assigned to DR-HC and those who were assigned to thrice-daily hydrocortisone. The numbers of AEs decreased during the first month of stage 2 when all patients received DR-HC.

Visits during stage 3 occurred every 6 months (patients who had not participated in stages 1 and 2 (*n*=16) had an additional 3-month visit). A summary of AEs that occurred during the first 18 months of stage 3 is given in Table 3. The percentage of patients with at least one AE during the first, second or third 6-month period of the 18-month follow-up ranged from 64.7 to 74.6%. The most common AEs were nasopharyngitis, fatigue, gastroenteritis, headache, vertigo, pyrexia, and arthralgia. The causality to treatment was scored as probably, possibly and not related for four (5.6%), 27 (38%), and 245 (91.5%) events. The most commonly reported AE possibly or probably related to treatment was vertigo in seven patients and fatigue in nine patients.

Throughout stages 1 and 2, no patients discontinued treatment because of an AE. Three patients in stage 3 discontinued because of AEs (Table 3): one patient died following a fall, one patient discontinued because of a subarachnoid haemorrhage and one patient discontinued because of fatigue related to a common cold.

Daily doses of DR-HC remained stable throughout stages 1 and 2, but could have been modified during stage 3. Nine patients decreased the daily dose of DR-HC during stage 3, six patients increased the dose and four patients changed the dose and then reverted back to the original dose.

Compared with baseline (randomisation) in stage 1 and follow-up at 18 months in stage 2, there was a mean reduction in body weight, up to 1.4 kg (s.d. 3.9) (*P*=0.046), but no change in systolic blood pressure (−1±13 mmHg), diastolic blood pressure (−1±10 mmHg) and HbA1c (0.1±0.5).

### AEs and cortisol exposure

The cortisol exposure profile of DR-HC was different from that of thrice-daily hydrocortisone. In particular, cortisol exposure 6–12 h after intake of DR-HC was markedly lower than that during thrice-daily administration of hydrocortisone, and the total daily exposure was also lower with DR-HC (18).

**Figure 3:**
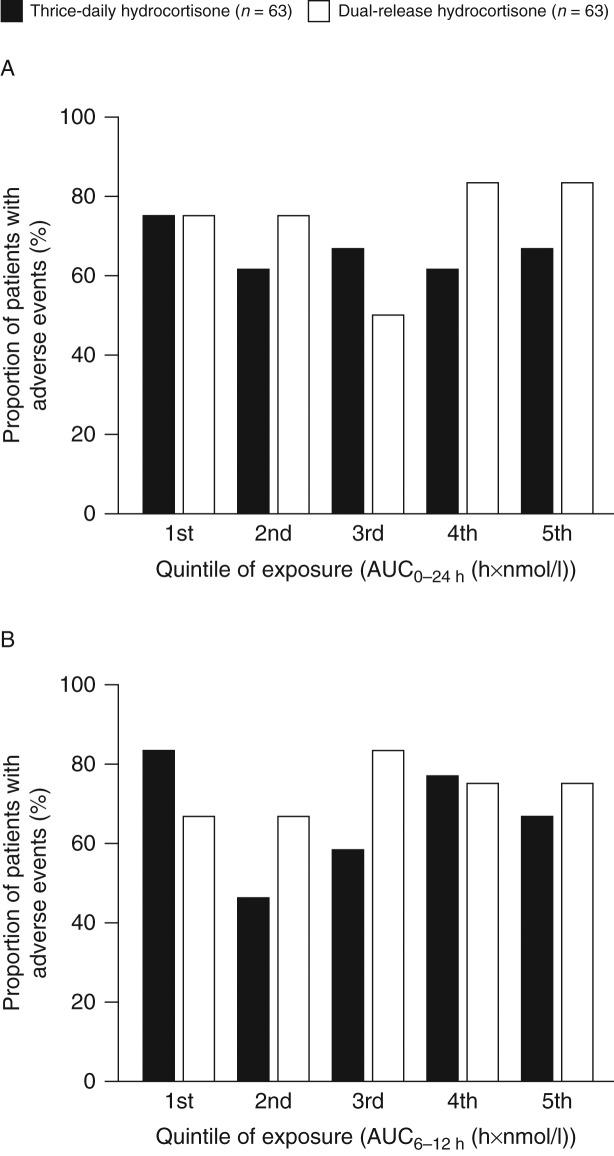
Percentages of patients with adverse events in quintiles of (A) total (AUC_0–24 h_) and (B) afternoon (AUC_6–12 h_) cortisol exposure; *P*>0.05 for comparisons of dual-release hydrocortisone (DR-HC) and thrice-daily hydrocortisone within each quintile. The quintiles for DR-HC were lower than for thrice-daily hydrocortisone as demonstrated by the median (min; max) values for AUC_0–24 h_ of 3844.7 h×nmol/l (1896.8; 7621.5 h×nmol/l) vs 4672.0 h×nmol/l (2658.3; 7867.2 h×nmol/l) and AUC_6–12 h_ of 785.1 h×nmol/l (213.4; 2289.4 h×nmol/l) vs 1551.7 h×nmol/l (927.8; 3050.9 h×nmol/l). AUC, area under the curve.

The number of patients with AEs in each quintile of total serum cortisol exposure (AUC_0–24 h_) was determined. There was no association between percentage of patients with AEs and total cortisol exposure both when patients received thrice-daily hydrocortisone and DR-HC (Fig. 3A). No statistically significant differences were observed between the treatments (*P*≥0.05 for all comparisons of DR-HC vs thrice-daily hydrocortisone). There was also no association between the frequency of AEs and cortisol exposure 6–12 h after administration of DR-HC, the period of the day when patients receiving DR-HC had lower cortisol exposure than those receiving thrice-daily hydrocortisone (Fig. 3B). The highest quintile of cortisol exposure during this period (>1097 h×nmol/l) corresponded to the lowest quintile during treatment with thrice-daily hydrocortisone (<1099 h×nmol/l). Thus, even though cortisol exposure while patients received DR-HC was lower than while patients received thrice-daily hydrocortisone, the level of exposure was not related to the rate of AEs.

## Discussion

This is the largest and longest prospective trial of the safety of glucocorticoid replacement therapy in patients with primary AI (Addison's disease). The study evaluated the safety of a newly developed DR-HC and showed that long-term maintenance treatment and rescue therapy were well tolerated.

By changing the cortisol-time profile, the DR-HC formulation resulted in an average 20% lower cortisol exposure than thrice-daily hydrocortisone, with 31% less exposure between 4 and 10 h after administration (1200–1800 h) and 59% less exposure between 10 and 24 h (1800–0800 h) (18). If the increase in AEs observed in stage 1 was related to reduced cortisol exposure, patients with the least exposure to DR-HC would be expected to have the highest incidence of AEs. The results showed no association between total or afternoon to evening cortisol exposure and the incidence of AEs. Of note, in the afternoon, the highest quintile of cortisol exposure when patients received DR-HC corresponded to the lowest quintile of exposure when patients received thrice-daily hydrocortisone, demonstrating the large difference between the two regimens in the cortisol profile. During all stages of the study, patients were aware of the treatment they were receiving, even in the randomised, crossover periods of stage 1. The increased incidence of AEs during stage 1 may reflect a vigilance of both patients and clinicians to monitor AEs while receiving therapy in the context of a clinical trial. In addition, there may have been a psychological impact of changing an established treatment regimen, which has been previously described in patients with AI (20, 21).

Patients with AI are in need of additional hydrocortisone during periods of stress, and underexposure to cortisol would be especially evident during periods of intercurrent illness. The mean number of intercurrent illness episodes per patient, mean number of days per episode, and mean dose per episode were similar between treatments in stage 1, suggesting that DR-HC is at least as effective as thrice-daily hydrocortisone therapy at controlling intercurrent illness. As patients continued to receive DR-HC during stages 2 and 3, the number of intercurrent illness episodes and the dose of hydrocortisone per episode remained relatively stable.

This study is the first to evaluate the management of intercurrent illness prospectively in patients with primary AI using a patient diary. Patient diaries were reviewed at each visit and increases in daily hydrocortisone dose due to intercurrent illness events were recorded. This information was also used to document any AEs and serious AEs according to standard criteria. However, this methodology would be likely to capture more intercurrent illness events than previous retrospective epidemiological studies of patients with AI, which have specifically defined adrenal crises as any impairment in general health that required intravenous glucocorticoids and hospitalisation (1).

The safety results of this study are based on 144.9 patient-years of exposure to DR-HC treatment. The AE profile and low withdrawal rates of patients receiving DR-HC indicate that the treatment was well tolerated. Overall, the most common AEs during the 18-month stage 3 follow-up period were nasopharyngitis, fatigue, gastroenteritis, headache, vertigo, pyrexia, and arthralgia. Persistence with DR-HC administered once daily was high, with only three patients discontinuing because of AEs. In addition, most patients who started in stage 1 of the study chose to continue in subsequent stages, which may reflect the high level of patient preference for DR-HC vs thrice-daily hydrocortisone, as previously reported (18). Most serious AEs during DR-HC treatment were infections leading to hospitalisation. Patients with primary AI are susceptible to infections according to previous register-based studies (22), which results in greater use of antimicrobial agents and increased hospital admissions related to infection compared with controls. In clinical practice (in Sweden), patients with AI are instructed to report to the hospital at the first sign of a gastrointestinal infection in order to treat or prevent a potentially dangerous state of acute AI, which explains why some patients were hospitalised for infections.

The main limitations of this study are the open-label administration of treatments in stage 1 and the lack of a comparator for DR-HC in stages 2 and 3. Primary AI is a rare disease, which limits patient numbers available for study. Also, as this is the only available prospective study on long-term safety of patients with primary AI, there is no possibility to perform valid comparison with long-term conventional replacement therapy. The strength of the data is, however, the collection of all events by conventional AE reporting as well as collection of data on increased use of hydrocortisone through patient diaries.

In conclusion, patients with primary AI receiving DR-HC therapy demonstrated a strong adherence to treatment that was well tolerated up to 27 months of continuous treatment. The short-term safety profile was similar to that of conventional thrice-daily hydrocortisone, and long-term safety remained stable both in terms of reported AEs and increased hydrocortisone use due to intercurrent illnesses. There was also a small reduction in body weight and no detrimental effects on blood pressure and glucose metabolism during long-term treatment with DR-HC.

## Author contribution statement

All authors contributed equally to this publication.

## Figures and Tables

**Table 1:** Patient demographics and baseline characteristics.

	**Patients initiating at the start of**	
Stage 1 (*n*=64)	Stage 3 (*n*=16)
Age, mean±s.d. (years)	47.2±13.6	45.6±13.1
Male, *n* (%)	37 (57.8)	5 (31.3)
Weight, mean±s.d. (kg)	79.4±14.3	70.8±13.2
BMI, mean±s.d. (kg/m^2^)	26.2±3.9	24.5±4.8
Systolic BP, mean±s.d. (mmHg)	123.5±19.5	119.5±14.8
Diastolic BP, mean±s.d. (mmHg)	75.9±11.4	73.8±7.2
Heart rate, mean±s.d. (beats/min)	65.2±10.5	61.5±9.3
Normal ECG, *n* (%)	57 (89.1)	16 (100)
Tobacco use, *n* (%)	11 (17.2)	1 (6.3)
Mean daily dose at run-in, *n* (%)		
20 mg/day	8 (12.5)	5 (31.3)
25 mg/day	7 (10.9)	5 (31.3)
30 mg/day	37 (57.8)	3 (18.8)
35 mg/day	0	2 (12.5)
40 mg/day	12 (18.8)	1 (6.3)

BP, blood pressure; ECG, electrocardiogram.

**Table 2:** Number of intercurrent illness episodes and days with increased usage of hydrocortisone during different stages of the study.

	**Patients recruited at stage 1** ^a^						**Patients recruited at stage 3**	
	Stage 1		Stage 2		Stage 3		Stage 3	
	Thrice-daily hydrocortisone	DR-HC	DR-HC		DR-HC		DR-HC	
0–3 months (*n*=64)	0–3 months (*n*=64)	3–6 months (*n*=59)	6–9 months (*n*=57)	9–12 months (*n*=55)	12–15 months (*n*=54)	0–3 months (*n*=16)	3–6 months (*n*=14)
Number of episodes per patient								
Mean (s.d.)	1.82 (1.67)	2.15 (1.87)	1.62 (0.65)	1.86 (1.29)	1.53 (0.87)	1.09 (0.30)	2.13 (1.13)	3.80 (4.09)
Median (range)	1.0 (1.0–8.0)	1.5 (1.0–9.0)	2.0 (1.0–3.0)	1.0 (1.0–5.0)	1.0 (1.0–3.0)	1.0 (1.0–2.0)	2.0 (1.0–4.0)	1.0 (1.0–10.0)
Number of days per episode								
Mean (s.d.)	3.30 (4.46)	2.44 (1.60)	2.76 (2.07)	2.94 (2.01)	2.37 (1.41)	4.64 (6.09)	2.28 (0.94)	2.91 (1.54)
Median (range)	2.0 (1.0–20.0)	2.0 (1.0–8.0)	2.0 (1.0–7.0)	3.1 (1.0–7.0)	2.0 (1.0–6.0)	3.0 (1.0–22.0)	2.0 (1.5–4.5)	2.8 (1.0–5.0)
Additional dose per episode (mg)								
Mean (s.d.)	17.65 (13.51)	22.84 (9.82)	19.62 (10.50)	16.38 (9.37)	20.83 (8.24)	14.85 (12.64)	26.11 (14.51)	19.74 (12.59)
Median (range)	10.0 (0–45.0)	20.0 (5.0–40.0)	20 (5.0–40.0)	14.0 (5.0–40.0)	20.0 (5.0–30.0)	11.7 (5.0–40.0)	21.7 (15.0–60.0)	20.0 (7.5–40.0)

DR-HC, dual-release hydrocortisone; NA, not available.

a Stage 1 was a crossover study in which patients were randomized to receive either thrice-daily hydrocortisone or DR-HC for 3 months before switching therapy for 3 months.

**Table 3:** Summary of adverse events during the first 18 months of stage 3; includes 55 patients who entered stage 3 from stages 1 and 2 and 16 new patients.

	**0–6 months** (*n*=71)		**6–12 months** (*n*=68)		**12–18 months** (*n*=68)		**0–18 months** (*n*=71)	
Events	Patients with ≥1 event, *n* (%)	Events	Patients with ≥1 event, *n* (%)	Events	Patients with ≥1 event, *n* (%)	Events	Patients with ≥1 event, *n* (%)
Adverse events	136	53 (74.6%)	79	44 (64.7%)	107	50 (73.5%)	322	68 (95.8%)
Serious adverse events	6	6 (8.5%)	4	3 (4.4%)	5	4 (5.9%)	15	10 (14.1%)
Discontinuation because of an adverse event	2	2 (2.8%)	0	0	1	1 (1.4%)	3	3 (4.2%)
Adverse events occurring in ≥10% of patients during 18 months of follow-up								
Nasopharyngitis	28	16 (22.5%)	18	14 (20.6%)	21	15 (22.1%)	67	31 (43.7%)
Fatigue	5	5 (7.0%)	6	6 (8.8%)	11	6 (8.8%)	22	15 (21.1%)
Gastroenteritis	11	10 (14.1%)	3	3 (4.4%)	5	5 (7.4%)	19	15 (21.1%)
Headache	11	11 (15.5%)	5	3 (4.4%)	2	2 (2.9%)	18	14 (19.7%)
Vertigo	7	6 (8.5%)	2	2 (2.9%)	3	3 (4.4%)	12	10 (14.1%)
Pyrexia	4	4 (5.6%)	3	3 (4.4%)	3	3 (4.4%)	10	9 (12.7%)
Arthralgia	1	1 (1.4%)	3	3 (4.4%)	5	5 (7.4%)	9	9 (12.7%)
